# Salutatory for 1856

**Published:** 1856-01

**Authors:** 


### HALL'S JOURNAL OF HEALTH.

OUR LEGITIMATE SCOPE IS ALMOST BOUNDLESS: FOR WHATEVER BEGETS PLEASURABLE AND HARMLESS FEELINGS, PROMOTES HEALTH; AND WHATEVER INDUCES DISAGREEABLE SENSATIONS, ENGENDERS DISEASE.

VOL. III.] JANUARY, 1856. [NO. I.

### SALUTATORY FOR 1856.

A HEALTHY New Year to you, reader! and as many returns of the same, as you will be entitled to by the moderate use of *all* the good things of this life, and a wise avoidance of the evil. And remember, that it is for yourself to determine, whether your aggregate conduct for the year eighteen hundred and fifty-six, shall be as *wood*, *hay*, *stubble*, in the great structure which is to lift humanity from want and sin, and raise it to the skies, or whether it shall be a grain of sand, a key-stone or a corner.

